# A Case of Amphetamine and Methamphetamine Intoxication in Cat

**DOI:** 10.3390/toxics10120749

**Published:** 2022-12-01

**Authors:** Agnieszka Chłopaś-Konowałek, Kaja Tusiewicz, Olga Wachełko, Paweł Szpot, Marcin Zawadzki

**Affiliations:** 1 Institute of Toxicology Research, 45 Kasztanowa Street, 55-093 Borowa, Poland; 2 Department of Forensic Medicine, Wroclaw Medical University, 4 J. Mikulicza-Radeckiego Street, 50-345 Wroclaw, Poland

**Keywords:** amphetamine intoxication, domestic animal intoxication, veterinary toxicology, stimulants, amphetamine, methamphetamine

## Abstract

Stimulants belonging to the amphetamine group nowadays pose an undeniable worldwide threat to the life and health of users. Intoxications of domestic animals also occur, which can either be accidental or related to intentional human action. This study presents the first ever reported case of a simultaneous amphetamine and methamphetamine intoxication of a cat, along with the results of toxicological studies. Blood, urine, vitreous humor and liver were collected during the cat’s autopsy and analyzed by UHPLC─QqQ─MS/MS. The sample preparation technique was based on one-step precipitation of proteins with cold acetonitrile. The determined amphetamine concentrations in the collected biological materials were 93.4 ng/mL in blood, 496.6 ng/mL in urine, 589.2 ng/mL in the vitreous humor and 291.2 ng/g in liver, respectively. Methamphetamine concentrations were 45.5 ng/mL in blood, 263.1 ng/mL in urine, 351.2 ng/mL in vitreous humor, and 97.7 ng/g in liver. Other substances were also found in the biological material, i.e., diazepam, oxazepam and nordiazepam. Cases of intentional or accidental poisoning of pets with psychoactive substances are a serious problem, carrying the risk to the health and life of the animal. Therefore, it is important to increase awareness of the high risk of poisoning of domestic animals, as well as to learn about the incompletely understood mechanisms of pharmacokinetics of various drugs in animals, including cats.

## 1. Introduction

Amphetamine, methamphetamine and other amphetamine-type stimulants are nowadays an undeniable worldwide problem and continue to pose a threat to human health. According to the United Nations Office on Drugs and Crime (UNODC) report [1], 79 tons of amphetamine and 325 tons of methamphetamine were seized globally in 2019. In Europe, the data are provided by the European Monitoring Centre for Drugs and Drug Addiction (EMCDDA). The report published in 2021 [2] indicates 17 tons of amphetamine and 2.9 tons of methamphetamine seized in 2019. In the same year, 27 million people worldwide are believed to have used amphetamine or methamphetamine [1], with about 30% doing so daily [2].

Illicit drug use is common and can lead to accidental or intentional intoxication of domestic pets. Exposure usually occurs via the oral or dermal route or by inhalation [3]. Janczyk et al. [4] described 213 cases of marihuana intoxications in dogs, where the majority of them were exposed to loose leaves or cigarettes, but in 10 cases the drug was present in a form of cookies. Drugs are the most common type of xenobiotics to which pets are exposed [5]. At the Kansas State Veterinary Diagnostic Laboratory, out of all drug-related cases involving cats and dogs, drugs accounted for 35%, with non-steroidal anti-inflammatory drugs being the most common. However, almost 4% of reported cases was represented by ADHD medications [3] which are known to contain amphetamine (e.g., Adderall^®^), methamphetamine (e.g., Desoxyn^®^) and other stimulants (e.g., Metadate^®^ with methylphenidate) as active ingredients [6]. The Animal Poison Control Center’s (APCC) database [7] information from 2010 to 2018 depicts more than 5000 intoxication cases involving amphetamine and methylphenidate in dogs and cats. Out of all cases, 4189 (83%) comprised dogs, whereas cats were involved in 844 (17%) reported cases. It is also worth noting that when illicit amphetamine or methamphetamine comes from the dark marketplaces and an unknown origin it may contain impurities that are, for example, a result of a synthesis process [8,9,10,11]. This could pose an additional risk both to the person using illicit drugs, but also to the pets that are unintentionally or intentionally exposed to a xenobiotic.

This paper aims to present the first ever reported case in the literature of a simultaneous amphetamine and methamphetamine intoxication in a cat.

### A Case Report

A 4-year-old European breed cat was adopted. However, two months later the temporary owner returned the cat to the shelter due to the fact that her partner probably had given the cat “some drugs” and had kicked the cat. The information sheet from the veterinary clinic showed that the owner, upon returning home, found the cat lying on the floor under the radiator. The cat was hypothermic and had peed on itself. The cat was then found to have abrasions on its back and on its left ear. The veterinarian also noted dilated pupils, very poor response to sounds, uncontrolled limb movements and the cat being unresponsive. The cat’s temporary owner took the cat to the shelter immediately after a visit in the veterinary clinic. According to the report of the caretakers, the cat was very weakened, lethargic, could not stand up, only laid on its side or crawled pushing off with its hind paws. During its stay at the shelter, the cat exhibited tachypnea and was by turns hypothermic and having a fever. The cat’s death occurred on the fourth day after being brought to the shelter. The cat’s owner pledged to contact the police.

## 2. Materials and Methods

### 2.1. Chemicals and Reagents

Water (Chromasolv^®^ LC–MS), acetonitrile (Chromasolv^®^ LC–MS), and formic acid were purchased from Sigma-Aldrich (Steinheim, Germany); ammonium formate was purchased from Sigma-Aldrich (Bangalore, India); amphetamine, methamphetamine, amphetamine-*d*_11_ (IS), methamphetamine-*d*_5_ (IS), diazepam-*d*_5_ (IS) were purchased from Cerilliant (Round Rock, TX, USA).

### 2.2. Biological Material

Drug-free blank blood samples used for the development and validation of the method were obtained from Regional Blood Donation Center. Blank samples were screened before spiking to ensure that they were free from drugs. The deceased cat was secured and transported to forensic veterinary section which took place two days after its death. The vitreous humor, blood and urine were collected into tubes with sodium fluoride, while the liver fragment was secured into a clean, disposable plastic container. The samples were then brought to the laboratory in a transport container at a controlled temperature of +4 °C for routine toxicological analysis in accordance with the shelter’s order. In the laboratory, they were stored at +4 °C (blood, urine, vitreous humor) and −20 °C (liver) until toxicological analysis. Under these conditions, stimulants such as amphetamine and its derivative, methamphetamine, are stable [12], differing from some halogenated cathinones, i.e., e.g., 3-CMC, 4-CMC (structurally similar to amphetamine), which are unstable in biological material at +4 °C [13].

### 2.3. Sample Preparation

A total of 200 μL of liquid biological sample (blood, urine, vitreous humor) was transferred into 2-mL Eppendorf tube. Then, 20 μL of methanolic IS (internal standard) solution (amphetamine-*d*_11_ 500 ng/mL, methamphetamine-*d*_5_ 500 ng/mL, diazepam-*d*_5_ 500 ng/mL) was added. The sample was mixed with 0.5 mL of cold acetonitrile (kept on ice) and vortexed to precipitate the proteins. The samples were then centrifuged at 13,500 rpm at 4 °C for 10 min. Then, 100 μL of the clear supernatant was transferred into glass inserts of the autosampler vials and analyzed by UHPLC─QqQ─MS/MS. Liver tissues samples were homogenized using an Q55 sonicator (QSonica, Newtown, CT, USA). In order to homogenize the tissue samples, 0.5 g of the solid specimen was transferred to a plastic tube (12 mL) and mixed with 0.5 mL of water (Chromasolv^®^ LC–MS). The tube was placed in a glass beaker containing ice cubes. Tissues were disrupted by the use of ultrasonic probe (5 kHz frequency). Next, 200 µL of the homogenate was subjected to the same procedure as the liquid samples.

### 2.4. Chromatographic and Mass Spectrometry Conditions

Analysis was performed using an ultra-high-performance liquid chromatography coupled with tandem mass spectrometry method (UHPLC─QqQ─MS/MS). Chromatographic separation was carried out with the use of a Kinetex^®^ XB-C18 column (150 × 2.1 mm i.d., particle size 2.6 μm; Phenomenex, Torrance, CA, USA). The mobile phase consisted of 0.1% formic acid and 10 mM ammonium formate in water (A) and 0.1% formic acid in acetonitrile (B). The gradient (at a constant flow of 0.4 mL/min) applied was as follows: 0 min, 5% B; 12 min, 98% B; 14 min, 98% B; and 15 min, 5% B. Return to the initial gradient compositions was performed for 5 min. The injection volume was 2.0 μL. Detection was achieved using a triple quadrupole mass spectrometer (Shimadzu 8050, Kyoto, Japan) equipped with an electrospray ionization (ESI) source. Determination of the substances was carried out in the multiple reaction monitoring (MRM) mode. The following MS parameters were fixed: nebulizing gas flow, 3 L/min; heating gas flow, 10 L/min; interface temperature, 250 °C; desolvation line temperature, 200 °C; heat block temperature, 350 °C; and drying gas flow, 10 L/min. A summary of precursor, product ions, collision energies, Q1–Q3 pre bias voltages and retention time for each compound are presented in Table 1.

### 2.5. Method Validation

The linearity, coefficient of determination (R^2^), lower limit of quantification (LLOQ), precision, accuracy, recovery, matrix effect and process efficiency were determined for blood. Those parameters were determined in the same way as those outlined by Szpot et al. [14]. Intraday precision and accuracy were evaluated in five replicates over one day and interday precision and accuracy were evaluated as one replicate over five subsequent days. Precision, accuracy, recovery, matrix effect and process efficiency were evaluated for amphetamine and methamphetamine at the concentrations of 10 and 100 ng/mL. Diazepam, nordiazepam were previously validated.

## 3. Results

Blood, urine, vitreous humor and liver specimens were collected for toxicological analysis. In all biological materials, amphetamine, as well as its homologue—methamphetamine were determined. Amphetamine concentrations were in the range of 93.4–589.2 ng/mL, whereas determined concentrations of methamphetamine were in the range of 45.5–351.2 ng/mL in all specimens. Moreover, diazepam, which is a pharmaceutical drug from the benzodiazepines class, was determined together with its metabolites: oxazepam and nordiazepam. All toxicological results are presented in Table 2.

Table 3 shows the validation parameters of the method. All presented values are in acceptable range for toxicological analysis of biological materials in accordance with GTFCh (German Society of Toxicological and Forensic Chemistry) recommendations, and thus, the method was implemented for routine toxicological analysis in our laboratory.

### Pathomorphological and Histophatological Findings

Postmortem and histopathological examinations did not reveal craniocerebral injuries or macroscopic or microscopic lesions that could have resulted from acute amphetamine and methamphetamine intoxication.

## 4. Discussion

Amphetamine and methamphetamine act as potent central nervous system (CNS) stimulants binding to the dopaminergic, noradrenergic and adrenergic receptors causing neurotransmitters’ release [15]. These phenylethylamine analogues’ activity depends on their chemical structure, e.g., methyl moiety added to the terminal amine group (methamphetamine vs. amphetamine) results in increasing the action on the CNS [16]. The most common symptoms of intoxication observed in animals include hypertension, tachycardia, hyperactivity, agitation, dilated pupils and seizures; however, lethargy and even coma were also reported [17]. Taking into consideration affecting CNS, amphetamine and its analogues easily penetrate blood-brain barrier and accumulate mainly in the grey matter structures of the brain, leading to inducing a so-called stereotyped behavior as a result of interaction with dopaminergic receptors in the nucleus caudatus [17]. Several studies are available that have looked into this topic more extensively. In 1966, Utena [18] studied behavioral symptoms resulting from methamphetamine administration on guinea pigs and mice. It was shown that among guinea pigs, methamphetamine caused a decrease in activity, while mice experienced agitation for about 4 h after administration of the xenobiotic, followed by a decrease in activity over the following days. A few years later, Ellinwood [19] studied stereotyped behavior in rats, cats and monkeys and described side-to-side head movements and repetitive sniffing, as well as increased attention towards surroundings. In other species, stereotypical behavior has manifested in the form of biting and chewing movements, examining fingers and hands (monkeys), as well as paws rubbing and abnormal posture positions (rats).

There are several papers describing cases of intoxication of domestic pets with psychoactive substances, such as the 213 dogs exposed to marihuana described by Janczyk et al. [4] or the case of intentional exposure of a cat to marijuana by its owner’s partner through smoke [20]. Cases involving poisoning with amphetamine and its analogues have also been reported in the literature [21,22,23,24]; however, data related to cats is very sparse [25] and thus the authors hope that the case described here may prove useful in adding to the knowledge on this topic. In all mentioned papers, dilated pupils and hyperthermia were observed in animals, which are typical sympathomimetic toxidrome’s symptoms. In this case report, the cat presented mydriasis, however, in comparison to others, its body temperature was low. This might result from the long time that could have passed before the owner found the cat lying on the floor. Pei et al. [21], as well as Diniz et al. [23] described the appearance of seizures in dogs intoxicated with methamphetamine and fenproporex (which metabolizes to amphetamine), however, in the case described, the authors have no information about the appearance of such an incident. Nevertheless, based on the substances detected in the biological material (diazepam and metabolites), the cat was administered drugs from the benzodiazepine group, which have a sedative but also anticonvulsant effect. The cat from the described case had a very poor response to sounds and later on it was unresponsive. Diniz et al. [23] also pointed out that restlessness might occur in amphetamine analogue intoxication, which eventually led to coma. However, it is worth noting that in the majority of cases, agitation, tachycardia, tachypnea and panting are observed [21,22,25]. A very intriguing aspect of amphetamine intoxication is related to a stereotypical behavior, which Wilcox et al. [22] described in their paper on a dog intoxication by Adderall^®^ pills. Eight hours after the dog was brought to the medical center it started circling to the right and paddling its hind limbs. Our paper presents a case of a cat which exhibited uncontrolled movements of the limbs, which is worth mentioning with regards to amphetamine intoxication.

To the best of authors’ knowledge, to this day no case of a simultaneous amphetamine and methamphetamine intoxication in cat has been reported. Only one paper by Crecraft et al. [25] described three cases of cats that were exposed to lisdexamphetamine, which is an active ingredient of ADHD medications. However, no concentration of this amphetamine analogue in biological material has been provided. Only one case on animal intoxication by methamphetamine includes xenobiotic’s concentration determined by gas chromatography coupled with mass spectrometry (GC−MS). Methamphetamine concentrations were 0.32 μg/mL and 2.35 μg/mL in dog’s serum and urine, respectively [21]. In this case report, that the authors believe is the first ever reported simultaneous amphetamine and methamphetamine intoxication in a cat, psychoactive substances were determined in four biological specimens. The highest concentrations of amphetamine and methamphetamine were present in urine (496.6 ng/mL and 263.1 ng/mL, respectively) and vitreous humor (589.2 ng/mL and 351.2 ng/mL, respectively). Moreover, diazepam with metabolites (in blood, urine and liver) were determined.

It is worth mentioning that the presence of both amphetamine and methamphetamine in all biological materials makes it difficult to determine what substance the cat was actually exposed to. The substance that the cat had been exposed to might have been a mixture of amphetamine and its analogue—methamphetamine. Amphetamine could have also been contaminated with methamphetamine accidentally, intentionally, or the methamphetamine may have been a residue from the synthesis process. However, the latter is unlikely due to the high and comparable to amphetamine concentration of methamphetamine in the biological materials. Moreover, it is also conceivable that the cat had previously been exposed to amphetamine for a long period of time and was eventually exposed to methamphetamine as well. Another possibility, however also unlike, is that it was pure methamphetamine that was metabolized into amphetamine. This is improbable due to the fact that about 4–7% of methamphetamine is metabolized to amphetamine in humans [26], and therefore the concentration of the latter should be much lower than observed in this case. Nevertheless, it should be emphasized that human and animals metabolism differ and moreover, it also differs between animal species. Thus, data related to humans should be interpreted with caution when taking into consideration cat’s intoxication. There are several papers raising the issue of amphetamine pharmacokinetics in animals, including cats.

A dose of 10 mg/kg of body weight administered intravenously in dogs can cause death, whereas LD_50_ for oral route of administration is estimated to be in the range of 20–27 mg/kg for amphetamine and 9–100 mg/kg for methamphetamine [6]. Both amphetamine and its analogue absorb rapidly through the gastrointestinal system. The onset of the clinical signs is 15–20 min for methamphetamine taken orally, whereas after ingestion, the peak plasma concentration for amphetamine occurs after 1–3 h in small animals [27]. Around 26% of amphetamine is transported in a form bounded to the plasma proteins in cats [28]. Amphetamine is rapidly distributed to the kidneys, liver, lungs, as well as adipose tissue and easily crosses the blood-brain barrier [27]. Latini et al. [17] observed that in cats, 0–5 min after amphetamine administration, the drug penetrated blood-brain barrier and was visible (due to the use of radiograms and isotope labeled amphetamine) mainly in the grey matter of the brain. The authors also examined plasma concentration of amphetamine in cats, stating 30 min as a time for distribution of this drug and then elimination with the half-time 510 min. Finally, Latini et al. [17] examined cats’ tissues amphetamine concentration, which resulted in values of the peak concentration in lungs, liver and spleen occurring 5 min after intravenous administration. Metabolism of amphetamines occurs in the liver mainly by hydroxylation and deamination (followed by oxidation and conjugation with glycine) pathways [27]. In humans, the metabolism of amphetamine and its analogues is regulated by cytochrome P450, mainly by its isoenzyme CYP2D6, with amphetamine exhibiting slightly greater affinity to this enzyme than methamphetamine [29]. Unfortunately, no data regarding isoenzymes related to amphetamines metabolism in cats is available, which only confirms the need for further research to expand our knowledge of amphetamines’ pharmacokinetics in animals. Amphetamine is eliminated in cats, dogs, chickens, rabbits, ponies and goats following first-order kinetics; however, in cats and dogs, the elimination seems to be much slower than in other mentioned species [28]. In dogs, amphetamine was eliminated within 6 h when their urinary pH was 7.5 and within 3.3 h when the urine’s pH equaled 6.0 [27], indicating that elimination of this xenobiotic to urine is pH dependent.

## 5. Conclusions

In this paper, to the best of authors’ knowledge, the first ever reported simultaneous amphetamine and methamphetamine intoxication in a cat was described. The high concentration of methamphetamine determined in the cat’s biological samples allows us to speculate that the cat was intoxicated by a mixture of amphetamine and methamphetamine. The cat exhibited the stereotypical behavior observed in amphetamine intoxication in animals. The described cases of poisoning of pets with psychoactive substances or drugs indicate a significant problem that should be handled with appropriate prevention. Despite the fact that several examples of poisoning of pets with psychoactive substances, including compounds from the amphetamines group, have been described, there is still very little knowledge about the pharmacokinetics of this group of compounds in animals, including cats. This indicates the need for further research and development of this issue. Moreover, the presented case explicitly demonstrates how important it is to complement clinical veterinary diagnostics with toxicological analysis and findings, as such an in-depth analysis of all the evidence can be useful in judicial proceedings.

## Figures and Tables

**Table 1:** Multiple reaction monitoring (MRM) conditions used in the ultra-high-performance liquid chromatography–tandem mass spectrometry (UHPLC–QqQ─MS/MS) analysis of substances and internal standards (IS).

Compounds	Retention Time [min]	Precursor Ions *(m/z)*	Product Ions *(m/z)*	Q1 Pre Bias [V]	Collision Energy [V]	Q3 Pre Bias [V]
Amphetamine	3.25	136.1	90.9 ^a^	−10.0	−21.0	−15.0
			65.1	−13.0	−39.0	−26.0
			119.2	−14.0	−15.0	−19.0
Amphetamine-*d*_11_	3.32	147.0	130.1 ^a^	−10.0	−15.0	−16.0
Methamphetamine	3.51	150.1	91.0 ^a^	−11.0	−21.0	−14.0
			119.2	−15.0	−16.0	−19.0
			65.0	−11.0	−39.0	−26.0
Methamphetamine-*d*_5_	3.49	155.0	91.0 ^a^	−14.0	−21.0	−15.0

^a^ ions selected for quantitative analysis.

**Table 2:** Concentrations of substances determined in biological materials from the authentic case.

Substance	Blood [ng/mL]	Urine [ng/mL]	Vitreus Humor [ng/mL]	Liver [ng/g]
Amphetamine	93.4	496.6	589.2	291.2
Methamphetamine	45.5	263.1	351.2	97.7
Diazepam	0.6	2.5	nd	5.3
Oxazepam	nd	3.7	nd	9.0
Nordiazepam	14.3	27.3	5.3	66.8

nd—not detected.

**Table 3:** Calibration curves parameters, LLOQ, recoveries, matrix effects, intra- and inter-day precision and accuracy of the UHPLC─QqQ─MS/MS method for determination of amphetamine and methamphetamine in blood.

Biological Matrix	The Linear Concentration Range [ng/mL]	The Coefficient of Determination (R^2^)	LLOQ [ng/mL]	Concentration Level [ng/mL]	Intraday		Interday		Recovery [%] *	Matrix Effect [%] *
					Precision RSD [%] *	Accuracy RE [%] *	Precision RSD [%] *	Accuracy RE [%] *		
AMPHETAMINE										
Whole blood	10–500	>0.9992	10	10	7	−7	1	−4	95	93
				100	6	−11	4	−13	91	92
METHAMPHETAMINE										
Whole blood	0.5–500	>0.9992	0.5	10	3	2	4	6	104	99
				100	7	6	2	12	99	93

* *n* = 5.
